# Inhibition of Notch4 Using Novel Neutralizing Antibodies Reduces Tumor Growth in Murine Cancer Models by Targeting the Tumor Endothelium

**DOI:** 10.1158/2767-9764.CRC-24-0081

**Published:** 2024-07-10

**Authors:** Jason W.-L. Eng, Yu Kato, Yusuke Adachi, Bhairavi Swaminathan, L.A. Naiche, Rahul Vadakath, Yoshimasa Sakamoto, Youya Nakazawa, Sho Tachino, Ken Ito, Takanori Abe, Yukinori Minoshima, Kana Hoshino-Negishi, Hideaki Ogasawara, Tomomi Kawakatsu, Miyuki Nishimura, Masahiko Katayama, Masashi Shimizu, Kazuhiro Tahara, Toshitaka Sato, Katsuhisa Suzuki, Kishan Agarwala, Masao Iwata, Kenichi Nomoto, Yoichi Ozawa, Toshio Imai, Yasuhiro Funahashi, Junji Matsui, Jan Kitajewski

**Affiliations:** 1 Division of Gastroenterology and Hepatology, Department of Medicine, College of Medicine, University of Illinois Chicago, Chicago, Illinois.; 2 Department of Physiology and Biophysics, College of Medicine, University of Illinois Chicago, Chicago, Illinois.; 3 Eisai Co., Ltd, Tsukuba, Japan.; 4 KAN Research Institute, Inc., Kobe, Japan.; 5 Eisai Inc., Woodcliff, New Jersey.; 6 University of Illinois Cancer Center, Chicago, Illinois.

## Abstract

Endothelial Notch signaling is critical for tumor angiogenesis. Notch1 blockade can interfere with tumor vessel function but causes tissue hypoxia and gastrointestinal toxicity. Notch4 is primarily expressed in endothelial cells, where it may promote angiogenesis; however, effective therapeutic targeting of Notch4 has not been successful. We developed highly specific Notch4-blocking antibodies, 6-3-A6 and humanized E7011, allowing therapeutic targeting of Notch4 to be assessed in tumor models. Notch4 was expressed in tumor endothelial cells in multiple cancer models, and endothelial expression was associated with response to E7011/6-3-A6. Anti-Notch4 treatment significantly delayed tumor growth in mouse models of breast, skin, and lung cancers. Enhanced tumor inhibition occurred when anti-Notch4 treatment was used in combination with chemotherapeutics. Endothelial transcriptomic analysis of murine breast tumors treated with 6-3-A6 identified significant changes in pathways of vascular function but caused only modest change in canonical Notch signaling. Analysis of early and late treatment timepoints revealed significant differences in vessel area and perfusion in response to anti-Notch4 treatment. We conclude that targeting Notch4 improves tumor growth control through endothelial intrinsic mechanisms.

**Significance:**

A first-in-class anti-Notch4 agent, E7011, demonstrates strong antitumor effects in murine tumor models including breast carcinoma. Endothelial Notch4 blockade reduces perfusion and vessel area.

## Introduction

Notch signaling directs cellular responses during normal development across an evolutionarily wide spectrum of organisms and is implicated in human disease pathogenesis. In mammals, four Notch proteins act as receptors for Notch ligands, and these regulate biological processes that range from cell fate determination to tumorigenesis (1). Notch1 to Notch4 share extensive homology within their extracellular domains (ECD), which consist primarily of EGF-like repeats linked to a transmembrane domain by a negative regulatory region (NRR; refs. 2–4). Receptor/ligand binding triggers proteolytic cleavage of the Notch receptors, releasing the intracellular domain (ICD) which translocates into the nucleus to drive transcription of downstream targets (4). The ICDs of Notch proteins exhibit variability outside of the conserved RAM domains and ankyrin-rich repeats; notably, Notch4 lacks an identifiable transactivation domain which is found in Notch1 (2).

Angiogenic sprouting and vessel maturation are guided by endothelial Notch proteins (5). Normal vascular endothelial cells (EC) principally express Notch1 and Notch4 (2). The critical role of endothelial Notch1 in angiogenesis arises from the regulation of endothelial tip and stalk cell identity, which facilitates the formation of nascent vessels (6). The role of Notch4 in angiogenesis has remained poorly defined. Global knockout mouse models suggest that Notch4 plays a proangiogenic role during development (7–9). However, varying reports describe antiangiogenic activity (10), overlapping activity with Notch1 (8, 9), or vessel diameter regulatory functions (11). Publicly available single-cell databases indicate that Notch4 expression is maintained in the adult endothelium in both mice and humans (12, 13). Notch4 expression increases in ECs of some mouse tumor models, including MMT-PyMT breast carcinomas (14) and ASV-B hepatocellular carcinoma (15). MMTV-PyMT–derived tumors implanted in Notch4-knockout mice exhibited reduced vessel perfusion and delayed onset of tumor growth, suggesting that Notch4 plays a distinct role in tumor endothelial function, although final tumor growth was not affected in this model (14). Interrogation of a public database of 26 human breast cancer samples shows that Notch4 is expressed primarily in ECs in tumors and further enriched in tip-like endothelial clusters (16). Bioinformatic analysis from human triple-negative breast cancer samples has identified endothelial Notch4 as a promising prognostic marker and highlighted its potential function in the tumor microenvironment (17).

To examine the role and therapeutic potential of Notch4 in tumor angiogenesis, we developed a first-in-class therapeutic blocking antibody against Notch4. By screening a hybridoma panel from an immunizing peptide consisting of the human Notch4 (hNotch4) extracellular EGF and NRR regions, we identified anti-Notch4, clone 6-3-A6, and created a humanized derivative, E7011. Treatment with 6-3-A6 or E7011 substantially reduced tumor growth and vessel function in multiple murine cancer models in which Notch4 was expressed in the tumor endothelium. Tumor models with low endothelial Notch4 expression responded poorly to Notch4 inhibition, even in cases in which tumor cells expressed Notch4. Using endothelium-specific, ribosome-associated mRNA sequencing to evaluate expression changes in a mouse breast cancer model treated with 6-3-A6, we determined that Notch4 inhibition alters tumor endothelial pathways associated with vascular function. Surprisingly, we did not observe an effect on downstream canonical Notch targets in response to 6-3-A6 treatment.

Antiangiogenic agents in single or combination therapy are the standard of care for many tumor types (18). However, most current antiangiogenic therapies target one common pathway, VEGF signaling, and many tumors develop resistance. Our results show that targeting Notch4 specifically alters perfusion and vessel area, contributing to improved tumor control in single or combination therapy; thus, these novel Notch4-blocking antibodies represent a powerful new tool for altering the tumor vasculature.

## Materials and Methods

### Cell lines

Calu-6, MDA-MB-231, DU145, Py8119, B16F10, and Lewis lung carcinoma cell lines were purchased from ATCC. The SEKI human melanoma cell line was purchased from JCRB. Py8119 cells were cultured in F12/K media (Gibco) with 10% FBS (Avantor) at 37°C with 5% CO_2_. B16F10 and Lewis lung tumor cells were cultured in DMEM (Gibco) with 10% FBS at 37°C with 5% CO_2_. All cell lines were tested for *Mycoplasma* weekly (MycoAlert, Lonza).

### Antibody development

Anti-Notch4 antibodies were developed by Eisai Co., Ltd. and KAN Research Institute. The hNotch4 EGFR27-29_NRR domain was purified as a fusion protein with secreted embryonic alkaline phosphatase and His10-tag from HEK293E cells (Invitrogen/Life Technologies) with Protino Ni 1000 prepacked columns and used to inoculate mice. Hybridomas were established by fusion of immunized mouse lymph node cells and P3U1 myeloma cells, and candidate antibodies were screened using the Notch4 Gal4-Luc assay described below. Clone 6-3-A6 was identified as anti-Notch4. Clone 6-3-A6 was humanized into E7011 by grafting the complementary-determining regions and selected framework residues of 6-3-A6 onto human antibody framework regions. The sequence encoding E7011 was inserted into the pcDNA3.3 vector (Invitrogen), transiently expressed using the FreeStyle 293-F system (Invitrogen), and purified from the culture supernatant with recombinant Protein A (GE HealthCare).

### Notch4-binding assays

To establish binding kinetics of E7011 and 6-3-A6, the rat anti–human δ-chain IgG was immobilized on a CM5 sensor chip. E7011 and human/mouse chimera anti-Notch4 (clone: 6-3-A6, hIgG1-chimera) were each captured on CM5 chips. Serial dilutions of recombinant Notch4 protein (from 0.31 to 20 nmol/L) were injected over the surfaces in multiple cycle kinetic experiments in Biacore A100. The flow rate was 30 µL/minutes, and association and dissociation times were 120 and 300 seconds, respectively. The double-referenced binding curves were fitted to the 1:1 Langmuir binding model using Biacore A100 evaluation software (GE HealthCare).

To determine Notch family specificity, human Notch1, Notch2, Notch3, and Notch4 were stably overexpressed in Raji, a human B-cell lymphoma cell line (D.S. Pharma). For overexpression of Notch4 derived from various species, such as human, mouse, and monkey, HEK293 cells were used. The cells were stained with control IgG2, E7011, or 6-3-A6 at 0.01 to 10 µg/mL for 20 minutes on ice, and after washing with 2% FBS in PBS, the cells were stained with PE-labeled anti–human or –mouse IgG antibody for 20 minutes on ice. Notch4 binding was determined by FACS using the FACSAria II and FACSymphony A5. The mean fluorescence intensity (MFI) was calculated using BD FACSDiva software and Cytobank.

### Notch4 Gal4-Luc assay

Using a vector expressing the Notch4 ECD::Notch1 ICD::Gal4 domain and a separate Gal4-UAS-Luc2CP vector, we established a stably overexpressing bEnd.3 mouse endothelioma cell line (ATCC) reporter cell line. Fifty microliters/well of 10 μg/mL recombinant human DLL4 protein (R&D) in PBS was poured in each well of a 96-well plate. After overnight incubation at 4°C, the 96-well plate was washed with PBS. Fifty microliters/well of 10^5^/mL of bEnd.3-Notch4 reporter cells and increasing concentrations of 6-3-A6 and E7011 were added in each well. The cells were cultured for 24 hours at 37°C. Using the dual-luciferase reporter assay system, firefly luciferase activity as a Notch signaling readout and Renilla luciferase activity as a control were detected by Envision.

### Xenograft, syngeneic tumor implantation, and nontumor studies

All procedures involving mice were approved by the Animal Care Committee at the University of Illinois Chicago, Health Science Center for Accreditation of Laboratory Animal Care, or conducted in accordance with the Institutional Animal Care and Use Committee guidelines of Eisai Co., Ltd., as appropriate for the location of mouse work performed. BALB/c*Foxn1*^*nu/nu*^ (nude) mice were obtained from the Charles River Laboratories, Japan. Cells were injected subcutaneously with 5 to 10 × 10^6^ cells in 100 mL of 1:1 mixture of Matrigel and culture media. 6-3-A6 or E7011 was diluted with PBS injected intravenously (i.v.) at a dosage of 25 mg/kg. Paclitaxel was diluted with ethanol/cremophor/5% glucose solution (1:1:18) and injected intravenously daily for 5 days. Bevacizumab was administered intravenously at 25 mg/kg twice weekly, and lenvatinib was given by oral gavage at 10 mg/kg daily. Tumor size was calculated as tumor volume (mm^3^) = long distance (mm) × short distance (mm) × short distance (mm)/2.

Male and female C57BL/6J mice were purchased from The Jackson Laboratory (Bar Harbor, ME, USA) and established in breeding colonies. For *in vivo* studies involving Py8119 tumor cells, a total of 10^6^ cells suspended in sterile 100 μL of PBS were orthotopically injected with a 27-g insulin syringe into the fifth mammary fat pad of 8- to 10-week-old female C57BL/6J mice. For *in vivo* studies involving B16F10 melanoma cells or Lewis lung carcinoma cells, a total of 5 × 10^5^ cells suspended in 100 μL of PBS were injected with a 27-g insulin syringe into the right lateral flank of 8- to 10-week-old male and female C57BL/6J mice. 6-3-A6 and E7011 were administered intravenously at 25 mg/kg twice weekly. For preventative studies, treatment was initiated 12 hours after tumor implantation. For treatment studies, tumors were allowed to reach ∼30 to 50 mm^3^ prior to beginning treatment.

For nontumor-bearing mice studies, male C57BL/6 mice were purchased and housed as above. E7011 or control IgG was administered intravenously at 25 mg/kg twice weekly for 4 weeks, and liver tissues were harvested. Images were photographed for gross examination.

### Vessel perfusion analysis

At indicated timepoints, nude mice bearing Calu-6 tumors were intravenously injected with Hoechst 33342 (0.5 mg/mouse) and then sacrificed after 5 minutes. C57BL/6 mice bearing Py8119 or B16F10 tumors were injected intravenously with fluorescent tomato lectin (VectorLabs, 50 μL/mouse) and sacrificed after 30 minutes. All tumor specimens were fresh-frozen at optimal cutting temperature and cryosectioned for analysis. To determine perfusion, tumor vessels were stained with anti-CD31 (BD Pharmingen). Hoechst-positive areas of each fluorescence image were determined by using Lumina Vision (ver. 2.2.2, Mitani Corporation), whereas tomato lectin–positive areas were analyzed using ImageJ software analysis. Perfusion was calculated as Hoechst-positive or tomato lectin–positive areas over the total CD31-positive area.

### Immunofluorescence and IHC staining

For immunofluorescence (IF) staining, tumors were harvested at indicated timepoints, bisected, frozen in optimal cutting temperature compound, and sectioned at a thickness of 7 to 10 μm. Prior to staining, the sections were air-dried, rinsed in PBS, and fixed in ice-cold acetone for 7 minutes. The sections were rinsed in PBS with 0.5% Tween-20 and then subsequently blocked with 3% normal goat serum in PBS-T to eliminate nonspecific binding.

Primary antibodies included CD31 (BD Pharmingen, 1:400), anti-CD68 (Thermo Fisher Scientific, 1:250), and Notch4 (Kitajewski lab, 1:250). Secondary antibodies included anti–rabbit Alexa Fluor 594 (Invitrogen, 1:1000), anti–rat Alexa Fluor 488 (Invitrogen 1:1000), and biotinylated goat anti-rat (VectorLabs, 1:500). All sections were incubated with primary antibodies diluted in blocking solution overnight at 4°C, washed, and incubated with secondary antibodies for 1 hour at room temperature. The sections were rinsed and mounted in VECTASHIELD with DAPI (VectorLabs) for IF. Images were acquired on a Zeiss Axio Imager at 10× magnification. Quantification of staining was performed using ImageJ software.

### Flow cytometry analysis

Single-cell suspensions from tumors were prepared by enzymatic dissociation with type IV collagenase (Worthington), 20 U/mL DNase I in DMEM, for 30 minutes at 37°C with agitation. The cells were strained through 100-μm and 70-μm meshes and then centrifuged for 30 minutes at 4°C at 1600 × *g* on a Ficoll cushion (Cytiva) to eliminate dead cells. Live cell counts were performed on a Countess II cell counter (Thermo Fisher Scientific), and a total of 2 × 10^6^ cells in 2 mL were used for staining per sample. Cells were blocked with 1 μL of anti-FC block followed by 0.5% BSA (Sigma-Aldrich) in PBS. Anti-CD31 (1 μL/10^6^ cells) and either E7011 (500 ng/10^6^ cells) or human IgG (500 ng/10^6^ cells) were added to each sample and incubated for 1 hour on ice, washed with ice-cold PBS, and incubated with anti–human Alexa Fluor 555 (Invitrogen) at 0.5 μL/10^6^ cells for 30 minutes on ice protected from light. The cells were washed and fixed with 1% methanol-free formaldehyde and stored at 4°C. Data were collected on BD FACSAria II and analyzed with FlowJo software. A total of *n =* 3 tumors/group were analyzed.

### EC RiboTag isolation and data analysis

RiboTag mice (19) were graciously provided for use by Peter Canoll (Columbia University, New York City, NY). Cdh5-Cre mice were obtained from the Ralf Adams Laboratory (20). *Cdh5-Cre*^*Tg/Tg*^*/RiboTag*^*flox/flox*^ (RiboTag^EC^) mice were maintained on a C57BL/6J background and genotyped by TransnetYX. RiboTag^EC^ female mice of 3 weeks old were intraperitoneally injected with tamoxifen dissolved in corn oil (75 mg/kg) for five consecutive days and rested until 8 weeks of age. Py8119 tumor cells were then implanted into the left fifth mammary fat pad, as previously described. Once tumors reached ∼30 mm^3^, the mice were randomized and then injected intravenously through the retro-orbital sinus with either 25 mg/kg of 6-3-A6 antibody or control mouse IgG (Jackson ImmunoResearch) twice weekly for 10 days. After the mice were euthanized, tumors were rapidly resected and snap-frozen in liquid nitrogen. Samples were stored at −80°C until polysome immunoprecipitation.

To isolate actively translating mRNA from EC polysomes, the tumor tissue was pulverized using a mortar and pestle in liquid nitrogen and suspended in RNA tissue lysis buffer [50 mmol/L Tris pH 7.5 (Invitrogen), 100 mmol/L KCl (Invitrogen), 15 mmol/L MgCl_2_ (Invitrogen), 100 μg of cycloheximide (Sigma-Aldrich), 1% Triton X-100 (Thermo Fisher Scientific), 1 mmol/L DTT (Sigma-Aldrich), 7.5 μL/mL protector RNA (Roche), 12 μL/mL Turbo DNase (Thermo Fisher Scientific), 10 μL/mL Halt Protease Inhibitor (Thermo Fisher Scientific), and RNAse-free H_2_O (Thermo Fisher Scientific)]. The mixture was transferred to a glass Dounce homogenizer on ice and then lysed with 10 strokes of pestle A, followed by an additional 10 strokes with pestle B. The lysed mixture was transferred to an ice-cold microcentrifuge tube and spun at 16000 × *g* for 10 minutes at 4°C. The supernatant was aspirated, and 100 μL was set aside for input analysis. The remainder of the supernatant was added to dynabeads (Thermo Fisher Scientific) conjugated to anti-HA antibodies (Abcam), and rocked on ice for 2 hours. The beads were separated by magnetic separation, washed twice with wash buffer, and then eluted using TRIzol (Thermo Fisher Scientific). The mRNA was isolated using a TRIzol Microprep kit (Zymo Research) and eluted into 13 μL of RNAse-free H_2_O. Prior to submitting the samples for sequencing, RNA quality and concentration were assessed using Agilent TapeStation 4200. Sequencing was performed on NovaSeq 6000 (SP flow cell, 100 bp single reads) at the DNA-sequencing laboratory at the University of Illinois at Urbana-Champaign.

Raw reads (*fastq*) were aligned with the mouse genome (mm10) using STAR (version 2.7.4a). The same files were processed to obtain raw counts using FeatureCounts (version 2.0.1), which was used as input for differential gene expression using DESeq2 (7). For visualization, normalized counts were adjusted to log scale (*rld*) and used to generate the principal component analysis (PCA) and heatmaps. To confirm that the IP fraction contained highly purified endothelial mRNA in immunoprecipitated (IP) fractions, we compared the enrichment of endothelial marker genes in the IP fraction with the original homogenate.

The initial PCA of the IP fraction showed low resolution between treated and control samples (*n* = 5 each), but after removing outliers, the separation between the two groups was more pronounced (PC1 = 60%, three controls, three treated). Differential gene expression analysis was performed using a design formula in DESeq2 (*design = ∼condition*), and changes in gene expression were assessed by log-fold changes (LFC). Genes with *P*_adj_ < 0.05 were identified as showing statistical differences in expression. LFCs greater than zero indicate upregulation, and LFCs less than zero indicate downregulation.

### qPCR analysis

For cDNA synthesis, a total of 2 ng of mRNA from the endothelial IP fraction was reverse-transcribed using the Verso cDNA synthesis kit (Thermo Fisher Scientific) with oligo-dT primers in a thermocycler (Applied Biosystems). A total reaction volume of 15 μL was loaded into a 96-well plate. Each reaction consisted of 200 pg of cDNA, 0.5 nmol/L of forward and reverse primers (IDT), 1x FastStart SYBR Green Master (Rox; Roche), and molecular-grade H_2_O. Reactions were amplified for 45 cycles on an ABI ViiA 7 qPCR system (Life Technology). Each sample was performed in duplicate. Calculation of the 2^−ΔΔct^ was performed using the formula Δct = average CT value of the gene of interest − the average CT value of β-actin. The ΔΔct was calculated by subtracting the Δct of the gene of interests from the average of the Δct from the treatment or control group and then calculating the 2^−ΔΔct^. *Hes1* primers (Forward 5′-CCA​GCC​AGT​GTC​AAC​ACG​A-3′; Reverse 5′-AAT​GCC‐GGG​AGC​TAT​CTT​TCT-3′). *Rnd1* Primers (Forward 5′-CAG​TTG​GGC​GCA‐GAA​ATC​TAC-3′; Reverse 5′-TGG​GCT​AGA​CTT​GTT​CAG​ACA-3′).

### Statistical analysis

Statistical analysis was performed as described in the figure legends unless otherwise specified. In figures without any notation, statistical analysis was performed and found not to be significant.

### Data availability

The data generated in this study are publicly available in Gene Expression Omnibus at GSE241629.

## Results

### Generation and characterization of first-in-class Notch4-blocking antibodies

**Figure 1:**
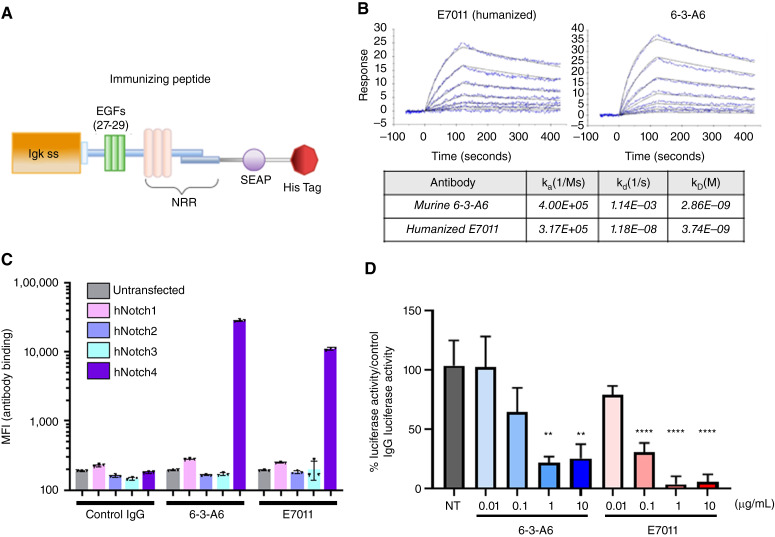
Development and validation of 6-3-A6 and E7011 as Notch4 inhibitors. **A,** Schematic of the immunizing epitope used to generate clone 6-3-A6. EGF, EGF repeats; Igk, immunoglobulin κ chain; SEAP, secreted embryonic alkaline phosphatase. **B,** Binding assay of the fully humanized E7011 and 6-3-A6 fused to human Fc for immobilization. Affinity to recombinant human Notch4 was calculated with surface plasmon resonance. **C,** Affinity of 6-3-A6 and E7011 to Raji cell lines generated to overexpress full-length human Notch1, Notch2, Notch3, and Notch4 (hNotch1 to 4, respectively). Binding was determined by MFI with flow cytometry analysis (*n* = 3). **D,** Luciferase reporter assay using a Notch4-ECD/Notch1-ICD-Gal4 fusion protein expressed in bEnd.3-immortalized ECs. Luminescence was measured with increasing concentrations of added E7011 or 6-3-A6. Statistical analysis performed with one-way ANOVA with the Dunnett multiple comparison test, plotted as average ± SEM; **, *P* < 0.01; ****, *P* < 0.0001.

The development of inhibitors specific to different Notch family members has been hindered by the high degree of shared homology in the ECD of the Notch proteins. To generate a Notch4-specific antibody, we targeted the NRR, a region of low homology between Notch proteins which has previously been used as the target for generating antibodies against Notch1 to Notch3 (21, 22). We fused human secreted embryonic alkaline phosphatase to the EGF-like repeats 27 to 29 of hNotch4 along with the NRR, followed by a 10xHis-tag sequence (Fig. 1A). The resulting recombinant protein was expressed and then isolated by His-tag purification and used to immunize mice. Hybridomas generated from immunized mice were screened for Notch4 reactivity, and clone 6-3-A6 was identified. The purified murine antibody 6-3-A6 was used as the basis for generation of a humanized version. We grafted the complementary-determining regions and framework residues of 6-3-A6 onto human antibody framework regions and generated the fully humanized antibody E7011.

To determine binding characteristics of these anti-Notch4 antibodies, we performed surface plasmon resonance (23). Anti–human IgG was immobilized onto chips to capture 6-3-A6 (fused to human Fc) or E7011, respectively. An analyte solution containing increasing concentrations of recombinant hNotch4 protein was flowed across the immobilized antibodies to determine association and dissociation constants. The binding curves and dissociation constants for 6-3-A6 and E7011 indicate high affinity binding for human, cynomolgus monkey, and mouse Notch4 with K_D_ values in the range of 1E^−10^ (Fig. 1B; Supplementary Fig. S1A). Because Notch family proteins share high degrees of homology, we validated the specificity of these antibodies by transfecting Raji lymphoma cell lines with full-length constructs for human Notch1, Notch2, Notch3, and Notch4 (Supplementary Table S1) and assessing binding of 6-3-A6 and E7011 by flow cytometry. MFI analysis demonstrated that both 6-3-A6 and E7011 bound specifically to human, cynomolgus monkey, and mouse Notch4 in a dose-dependent manner but not to other Notch proteins (Fig. 1C; Supplementary Fig. S1B).

We verified the ability of these antibodies to block Notch4 activation using an *in vitro* luciferase reporter system designed to measure proteolytic cleavage–dependent Notch transcriptional activity. bEnd.3-immortalized ECs were transfected with a fusion protein comprising the Notch4 ECD, the Notch1 ICD, and a C-terminal Gal4 domain. The Notch4–ligand interaction was assessed following the release of the cleaved ICD, which was detected using a cotransfected vector containing a luciferase reporter under the transcriptional regulation of the Gal4 upstream activation sequence. Dual-transfected cells were seeded onto DLL4-coated plates to induce Notch4 activation. Increasing concentrations of 6-3-A6 or E7011 reduced luciferase activity in a dose-dependent manner, indicating that both antibodies effectively inhibit DLL4-mediated Notch4 activation (Fig. 1D). In these experiments and subsequent *in vivo* testing in which 6-3-A6 or E7011 was examined, the activities of these two were statistically indistinguishable, allowing us to assess and describe the functions of both antibodies.

### Anti-Notch4 antibodies decrease tumor growth in human tumor xenograft models that express endothelial Notch4

**Figure 2:**
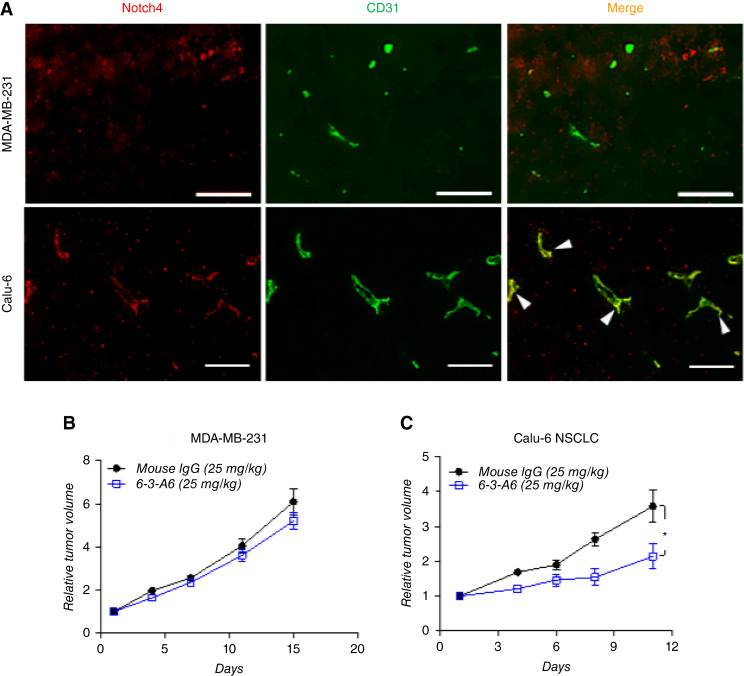
Treatment of human tumor xenografts with 6-3-A6 delays growth of tumors which express endothelial Notch4. **A,** IF staining of Notch4 (red) and CD31^+^ tumor vessels (green) in human tumor xenografts. Arrowheads indicate Notch4-expressing vessels (Scale bar = 200 μm). **B** and **C,** Tumor growth curves of subcutaneously implanted tumors from the indicated cell lines. **B,** MDA-MB-231 xenografts treated intravenously with 6-3-A6 (*n* = 5). **C,** Calu-6 human NSCLC xenograft tumors treated intravenously with 6-3-A6. All treatments started 12 hours after tumor implantation. Statistical analysis performed with two-way ANOVA for tumor growth curves, plotted as average ± SEM; *, *P* < 0.05.

To evaluate the function of tumoral Notch4, we first determined tumor cell and stromal cell types that express Notch4. Prior studies have indicated that Notch4 can be expressed by tumor endothelium, immune cells, and some cancer cells (24–27). We examined the presence of Notch4 by IF staining of tumors derived from Calu-6 non–small cell lung carcinoma (NSCLC) and MDA-MB-231 triple-negative breast carcinoma implanted into BALB/c nude mice. Although analysis of both tumor models identified detectable levels of Notch4, in Calu-6 xenografts, the Notch4 signal was primarily localized to CD31-positive ECs, whereas in MDA-MB-231 xenografts, Notch4 expression did not overlap with the tumor vasculature (Fig. 2A).

To evaluate 6-3-A6 efficacy against human xenograft tumor models, MDA-MB-231 or Calu-6 tumor cells were subcutaneously implanted into nude mice and treated intravenously with 6-3-A6 (25 mg/kg twice weekly) or control mouse IgG. We observed a significant decrease in the growth of Calu-6 tumors treated with anti-Notch4 therapy compared with control treatment (Fig. 2B). In comparison, treatment of MDA-MB-231 xenografts did not cause any growth delay (Fig. 2C). These findings suggest that endothelial Notch4 expression is crucial for tumors to respond to 6-3-A6 therapy.

**Figure 3:**
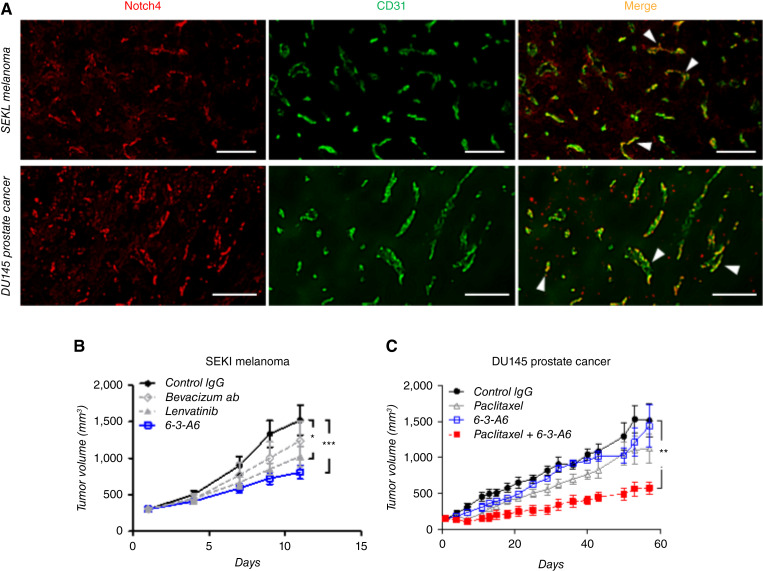
6-3-A6 treatment alone or in combination with other chemotherapeutic agents delays tumor growth in human tumor xenografts. **A,** IF staining of Notch4 (red) and CD31^+^ tumor vessels (green) in additional human tumor xenografts. **B,** SEKI melanoma human xenografts treated with 6-3-A6 alone, i.v. bevacizumab alone, or lenvatinib alone (*n* = 5). **C,** DU145 prostate xenografts treated with i.v. 6-3-A6, i.v. paclitaxel, or combination (*n* = 4 mice/group). All treatments started 12 hours after tumor implantation. Statistical analysis performed with two-way ANOVA for tumor growth curves, plotted as average ± SEM; *, *P* < 0.05; **, *P* < 0.005; ***, *P* < 0.001.

We identified additional human tumor xenografts that expressed endothelial Notch4 (Fig. 3A), including SEKI (melanoma) and DU145 (prostate cancer). Similar to Calu-6, SEKI melanoma tumors responded to treatment with 6-3-A6 (Fig. 3B). Using this model, we evaluated 6-3-A6 against clinically available antiangiogenic agents. In Calu-6 tumors, 6-3-A6 showed antitumor effects comparable with, or possibly stronger than, lenvatinib treatment, whereas bevacizumab did not significantly impact growth (Fig. 3B). In DU145 tumors, 6-3-A6 as a single agent did not affect tumor growth but significantly enhanced the efficacy of paclitaxel treatment (Fig. 3C). We conclude that anti–Notch4-blocking agents have potent antitumor effects similar to clinically available antiangiogenic therapies, which may enhance the efficacy of existing chemotherapeutics.

Previous preclinical studies with Notch or Notch ligand inhibitors revealed several adverse side effects, including induction of vascular neoplasms or weight loss (22). During our studies, we monitored body mass as an indicator of drug toxicity. In Calu-6, SEKI, and DU145 xenograft models, we saw no changes in the body mass of mice treated with 6-3-A6 compared with treatment with control IgG even up to 60 days of treatment (Supplementary Fig. S2A–S2C). In non–tumor-bearing C57BL/6 mice treated with E7011 or control IgG for 4 weeks, we performed a gross examination of the livers. Although prior studies have shown dilation of the sinusoids in the distal portions of the hepatic lobes in response to downstream Notch1 inhibition, we saw no overt changes to the vessels in response to E7011 (Supplementary Fig. S2D–E; ref. 28). These findings indicate that Notch4 blockade may be a safe therapeutic strategy.

### Anti-Notch4 antibodies reduce growth of syngeneic tumors

**Figure 4:**
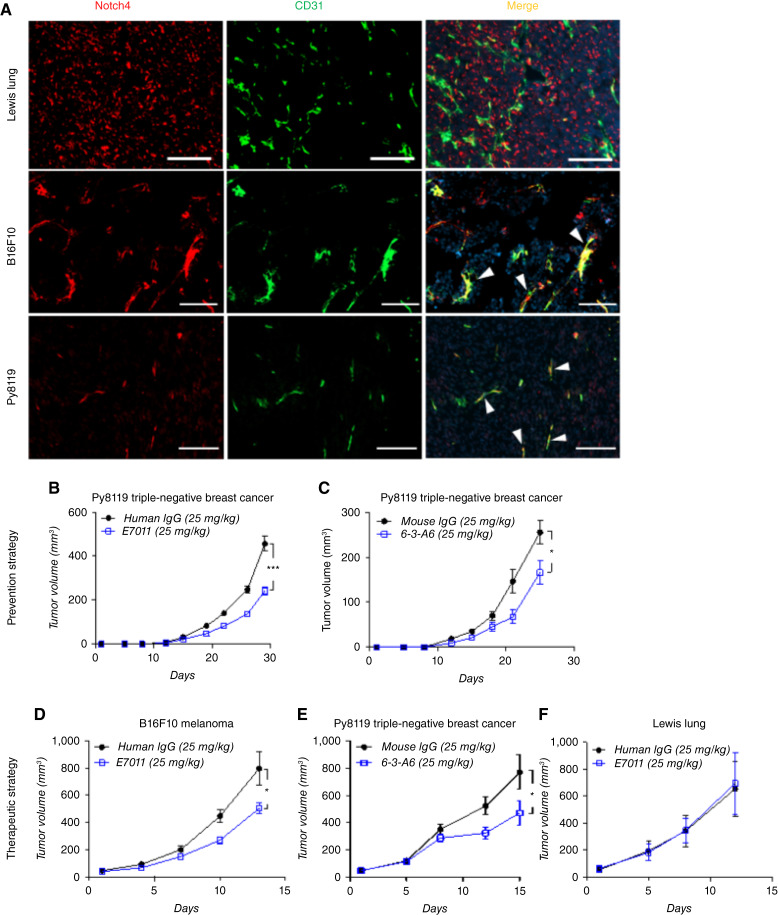
Treatment of tumors with 6-3-A6/E7011 delays tumor growth in syngeneic tumor models which express endothelial Notch4. **A,** IF staining of murine Lewis lung carcinoma, murine Py8119 breast carcinoma, and murine B16F10 melanoma with Notch4 and CD31. Arrowheads indicate Notch4-expressing vessels (scale bar = 200 μm). **B** and **C,** Preventative strategy using Py8119 tumor cells orthotopically implanted into the mammary fat pad and treated with **B,** E7011 (*n* = 18 human IgG, *n* = 17 E7011) or **C,** 6-3-A6 (*n* = 4) twice weekly starting 12 hours following tumor implantation. **D** and **F,** Tumor growth curve of subcutaneously implanted **D,** Lewis lung (*n* = 4) and **E,** B16F10 (*n* = 7) treated intravenously with E7011 (25 mg/kg twice weekly) and orthotopically implanted **F,** Py8119 murine breast carcinomas (*n* = 5) treated intravenously with 6-3-A6 (25 mg/kg twice weekly) once the average tumor volume of each cohort had reached approximately 30 mm^3^. Statistical analysis performed with two-way ANOVA for tumor growth curves, plotted as average ± SEM; *, *P* < 0.05; ***, *P* < 0.001. TNBC, triple-negative breast cancer.

To recapitulate the diversity of the tumor microenvironment, we studied orthotopically and subcutaneously implanted syngeneic tumor lines in mouse models. We analyzed tumors grown in immunocompetent mouse models using three C57BL/6J syngeneic tumor lines: Lewis lung carcinoma (NSCLC), Py8119 (mammary carcinoma cells arising from the polyoma middle T antigen–expressing transgenic murine model), and B16F10 (murine melanoma cells). As seen in the xenograft models, endothelial Notch4 expression varied among tumor types (Fig. 4A). Py8119 and B16F10 primarily expressed Notch4 in ECs, whereas Lewis lung tumors showed expression predominantly in nonvascular cell populations.

To evaluate the effect of anti-Notch4 treatment in tumors grown in immunocompetent mice, we implanted Lewis lung or B16F10 cells subcutaneously in the flanks of C57BL/6J mice or injected Py8119 cells orthotopically into the mammary gland of female C57BL/6J mice. We first assessed whether anti-Notch4 therapy could prevent tumor growth in preclinical models. Py8119 cells were implanted orthotopically into the mammary gland, and treatment was initiated 12 hours later with i.v. control human IgG or E7011. Early preventative treatment of Py8119 tumors with E7011 significantly reduced tumor growth and final tumor mass when compared with control IgG (Fig. 4B; Supplementary Fig. S2F). To ensure that the therapeutic response was not caused by a nonspecific murine reaction to the humanized antibody, we replicated these Py8119 experiments with murine 6-3-A6 and observed a similar degree of tumor growth inhibition as seen with E7011 (Fig. 4C).

To mirror a therapeutic approach available to patients, tumors were allowed to establish and grow to 30 mm^3^ before starting treatment. In these established tumors, 6-3-A6/E7011 inhibited further growth in B16F10 (Fig. 4D) and Py8119 (Fig. 4E), which expressed endothelial Notch4, but not in the Lewis lung carcinoma model (Fig. 4F). These findings support the importance of tumor endothelial Notch4 in mediating tumor growth control by 6-3-A6/E7011 treatment. Thus, anti-Notch4 treatment may have broad clinical benefits in different cancers at different stages.

### Notch4 blockade alters endothelial gene signatures associated with angiogenesis

**Figure 5:**
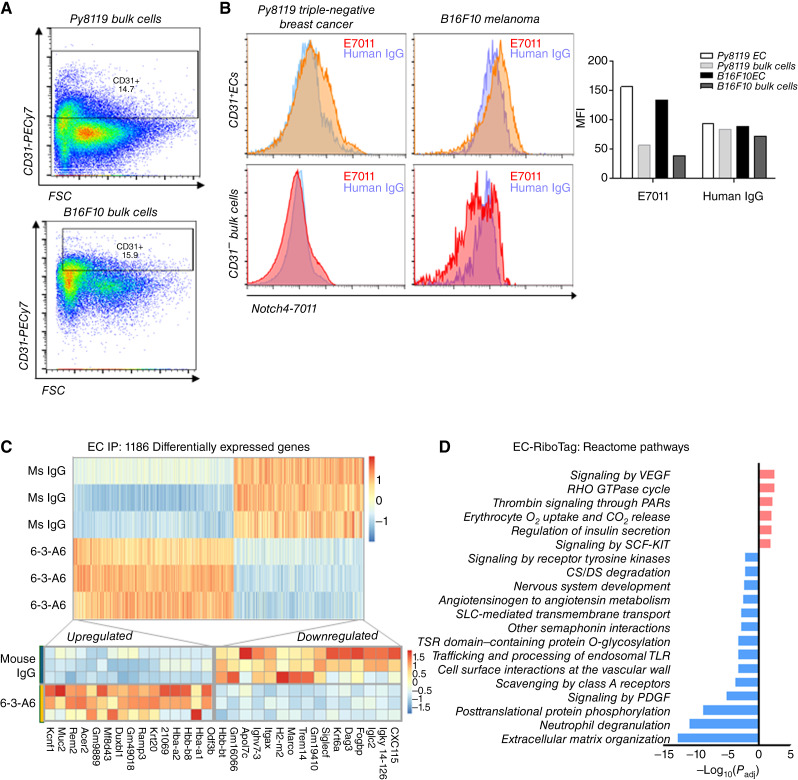
Notch4 is expressed on tumor ECs, and treatment alters the tumor vessel transcriptional profile. **A,** Representative flow plots of gating for CD31^+^ cells in Py8119 and B16F10 tumors. **B,** Histograms comparing E7011 and human IgG binding to CD31^+^ and CD31^−^ cell populations from dissociated Py8119 and B16F10 tumors (*n* = 3). Left, E7011 samples were concatenated and merged into a single representative histogram. Right, Graph of concatenated MFI from Py8119 and B16F10 samples bound with E7011 or human IgG. **C,** Heatmaps of significantly upregulated and downregulated differentially expressed genes isolated from IP polysomes from tumor ECs treated with 6-3-A6 or control IgG (*n* = 3). Boxes indicate genes implicated in GPCR control of angiogenesis (red), endothelial nitric oxide regulation (blue), and inflammatory/immune response (green). **D,** Reactome pathway analysis of upregulated and downregulated pathways was performed using g:Profiler, and significance threshold by Benjamini–Hochberg FDR was set at 0.05. **A,** Fold cutoff of −log_10_(*P*_adj_) > 2 was used to select the displayed pathways. Statistical analysis performed with the *t* test for perfusion studies, plotted as average ± SEM; *, *P* < 0.05; **, *P* < 0.005. Heatmap data presented as *Z*-scores, which integrate both fold change and significance.

Our findings using different tumor models indicated that endothelial Notch4 in tumor vasculature was critical for 6-3-A6/E7011 to restrict tumor growth. Using flow cytometry on dissociated tumor cells from syngeneic Py8119 and B16F10 tumors, we confirmed that E7011 bound to CD31^+^ ECs but not to the CD31^−^ stromal and bulk tumor cells (Fig. 5A and B). To evaluate signaling pathways mediating tumor endothelial Notch4 function, we conducted endothelial translatomic analysis using the *Rpl22*^*tm1.1Psam*^ (RiboTag) system, whereby we isolated cell type–specific polysome-bound mRNA (19) in combination with endothelium-specific *Cdh5*^*TgCreERT2*^, referred to as RiboTag^EC^ mice. Endothelium-specific RiboTag^EC^ expression was induced with tamoxifen by intraperitoneal injection (75 mg/kg for 5 days) in 3-week-old mice, which were then orthotopically implanted with Py8119 tumor cells at 8 weeks (Supplementary Fig. S3A). Once tumors reached an average of 50 mm^3^, 6-3-A6 or control IgG was administered, as above. Tumors were isolated 10 days after the start of treatment in order to minimize secondary effects resulting from differences in tumor size (Supplementary Fig. S3B) and to assess Notch inhibitory effects in early phases of tumor vascularization. HA-tagged endothelial ribosomes were immunoprecipitated directly from snap-frozen tumor homogenates (29). Sequencing of mRNA from total tissue homogenates and tagged ribosome-associated IP fractions confirmed high purity of endothelial mRNA isolation (Supplementary Fig. S3C). Subsequent PCA of the endothelial IP gene signatures revealed differential clustering of the control IgG and 6-3-A6 treatment groups (Supplementary Fig. S3D).

Differential gene expression analysis identified 1,186 significantly upregulated and downregulated genes (Fig. 5C). Among the most significantly elevated by 6-3-A6 were genes associated with GPCRs (G protein coupled receptors) involved in angiogenesis (*Ramp3* and *Rem2*) and hemoglobin genes (*Hbb-bt*, *Hba-a1*, *Hbb-bs*, and *Hba-a2*) critical to nitric oxide transport in ECs (30). Further analysis of all significant differential gene expressions using the Reactome database confirmed that pathways known to be suppressed by Notch signaling, such as VEGF, thrombin, and Rho pathways, were upregulated by anti-Notch4 treatment (Fig. 5C). However, when we inspected the expression of previously described Notch target genes to determine whether anti-Notch4 treatment affected canonical Notch signaling, neither translatomic analysis (Fig. 5C and D) nor qPCR validation of the IP mRNA (Supplementary Fig. S3E) identified any consistent changes in downstream canonical Notch targets.

Many of the upregulated pathways, such as VEGF signaling, included regulated genes more closely associated with EC function and vascular growth (*Bcar1/P130Cas*, *Hras*, *Pgf*, and *Nos3*), whereas downregulated pathways had much greater enrichment of genes related to the inflammatory response (*Marco* and *Tlr8/9*) and ECM remodeling (*Col6a3, Fn1*, and *Mmp9*; Fig. 5D; Supplementary Fig. S3F). These molecular pathways are closely linked to vessel perfusion and growth, indicating that vascular function may be altered in response to treatment. Moreover, the results suggest that Notch4 function and signaling differ substantially from those of Notch1, which suppresses tumor angiogenesis and activates canonical signaling.

### Anti-Notch4 treatment alters vessel density and vascular perfusion in tumor models

**Figure 6:**
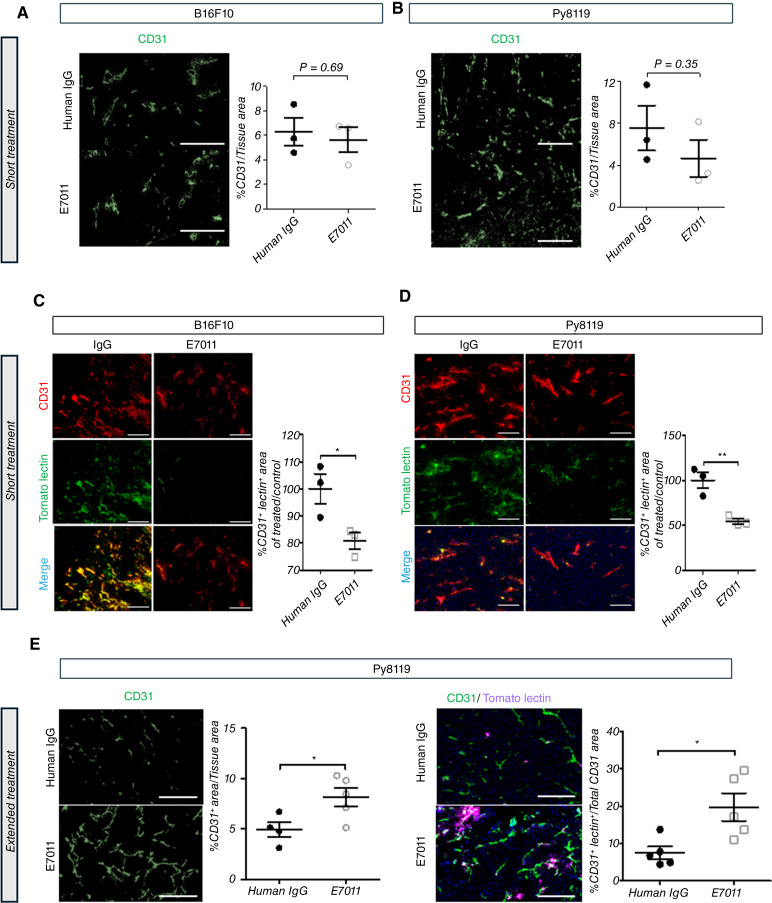
Anti-Notch4 treatment alters vessel perfusion and angiogenesis. **A** and **B,** Assessment of vessel coverage area by CD31^+^ staining in **A**, B16F10 melanoma treated for 4 days with two doses (days 1 and 3) of 6-3-A6 (*n* = 3) and **B**, Py8119 tumors treated for 10 days with four doses (days 1, 3, 6, and 9) of 6-3-A6 (*n* = 6). Right, Representative images; left, quantification of colocalization. (Scale bar = 100 μm). **C** and **D,** Assessment of tumor perfusion in syngeneic **C,** B16F10 melanoma (*n* = 3) and **D,** Py8119 tumors (*n* = 6) using intravenously injected fluorescent tomato lectin–Alex Fluor 488. Right, Representative images costained with CD31 (red); left, quantification of colocalization. (Scale bar = 100 μm). **E** and **F,** IF staining of **E,** CD31^+^ tumor vessels in orthotopically implanted Py8119 tumors treated for 4 weeks with E7011 (*n* = 4), and **F,** vessel perfusion by fluorescent tomato lectin–Alexa Fluor 647 labeling with CD31 in Py8119 tumors treated for an extended duration (*n* = 5). Right, Representative images; left, quantification of the staining or colocalization. (Scale bar = 200 μm). Statistical analysis by the *t* test, plotted as average ± SEM; *, *P* < 0.05.

To correlate the translatomic signature of tumor ECs with phenotypic changes in response to anti-Notch4 treatment, we assessed vascular growth and functional perfusion. In the Py8119 and B16F10 syngeneic tumor models, we observed no major differences in the percentage of area marked by CD31 (vessel area) after a short course of E7011 treatment; that is, before treatment induced significant differences in tumor size (Fig. 6A and B; Supplementary Fig. S4A and S4B). However, notable decreases in the perfusion of tumor vessels were observed in Py8119 and B16F10 after a short course of treatment (Fig. 6C and D). The ability of E7011 to reduce vessel perfusion was observed at all dosages tested, down to 1 mg/kg, demonstrating that Notch4 blockade efficiently disrupts tumor vessel function (Supplementary Fig. S4C–S4E). To evaluate whether these effects persisted, we analyzed Py8119 tumors after 4 weeks of treatment, once tumors had significantly diverged in size. We observed a significant increase in the vessel area of tumors treated with E7011 compared with control IgG (Fig. 6E). Intravenous tomato lectin injections highlighted increased vascular perfusion with areas of notable regions of extravasation into the extravascular space at this later timepoint (Fig. 6E). These findings indicate that long-term Notch4 blockade leads to hypervascularized tumors and potentially improved blood flow within tumors near experimental endpoints.

## Discussion

Notch signaling functions depend on cell type, timing of activation, and cross-talk with other signaling pathways. Tumor-promoting Notch signaling contributes significantly to a variety of solid and liquid tumor types; thus, Notch signaling proteins and associated Notch regulators have been the focus of therapeutic targeting. Previous efforts have focused on targeting γ-secretase, DLL4, and Notch1 to Notch3. Although both Notch1 and Notch4 are highly expressed on tumor ECs, only the effects of Notch1 signal inhibition have been intensively studied. Inhibition of Notch1 leads to hypersprouting and vessel dysfunction, increased hypoxia, and ultimately decreased tumor growth, but candidate therapeutics targeting Notch1 have exhibited significant toxicity in normal tissue (31, 32). Notch4 targeting is less explored; thus, we sought to establish if Notch4 inhibition has therapeutic potential. We provide compelling evidence that endothelial Notch4 promotes cancer development and alters the tumor vasculature. Notch4 inhibition affected tumor growth, tumor vessel function, and perfusion. We conclude that the development of a first-in-class mAb, E7011/6-3-A6, which specifically blocks Notch4 activity, provides a major therapeutic advance against tumor growth.

The generation of the Notch4-neutralizing antibody marks an important step forward in Notch research. Therapeutics targeting the Notch pathway, including γ-secretase inhibitors and blocking antibodies against Notch1 to Notch3, have previously been developed and demonstrated significant antitumor effects in preclinical studies. Unfortunately, these agents affect both neoplastic and normal tissues, particularly the gastrointestinal tract, skin, and brain, leading to a host of toxicities (33). Notch1-containing decoy peptides have been shown to block tumor growth with less toxicity than γ-secretase inhibitors; however, similar Notch4-containing peptides have not been evaluated against tumors (3, 34). Unlike the inhibition of other Notch proteins, Notch4 blockade does not seem to cause complications to tissue vasculature. In our preclinical models, we did not witness any adverse effects regularly encountered with other Notch-targeted therapies. These observations are consistent with mild phenotypes observed in Notch4 global knockout mice (7, 8, 14). Moreover, the high specificity of E7011/6-3-A6 for Notch4 likely mitigates any off-target binding to other Notch proteins in other tissues. Thus, inhibiting Notch4 activity could be a promising and safe avenue for future cancer treatment.

A variety of contradictory roles have been documented for Notch4 in angiogenesis; the most frequently documented phenotypes suggest that Notch4 acts to promote vascular outgrowth and increase vessel lumen size and perfusion (7–11, 14). In our study, the direct effects of Notch4 blockade on tumor vasculature via E7011/6-3-A6 treatment varied depending on the stage of tumor growth. At early stages after tumor implantation, the vascular area was not changed, but perfusion decreased because of Notch4 inhibition, consistent with prior literature. At late stages, vascular density increased in treated tumors along with increased vascular leakage, which is more consistent with Notch4 playing an antiangiogenic role. Whether these effects were due to direct ongoing inhibition of Notch4 signaling or a compensatory response to the loss of downstream Notch4 inactivation remains to be explored. Notably, the translatomic signature within the tumor ECs after a few days of treatment with 6-3-A6 revealed increased activity of VEGF signaling, suggesting that Notch4 signaling may attenuate angiogenesis in tumors and that the increase in vessel area observed after extended 6-3-A6/E7011 treatment may in fact be due to Notch4 inhibition. More detailed work is needed to examine the full extent of these changes in angiogenesis. For instance, Notch1 inhibition within tumor and the subsequent hypersprouting leads to hypoxia and tumor growth reduction (34). Whether changes in hypoxia are also involved in Notch4-mediated tumor inhibition remains to be explored.

Beyond VEGF signaling, we noted changes in inflammatory gene expression. A growing body of work has suggested that endothelial Notch4 may affect inflammation either directly in ECs or indirectly via angiocrine signaling. Notch4 null mice show reduced glomerular injury and cytokine response to human immunodeficiency virus–triggered inflammation (35). In humans, Notch4 triggers endothelial dysfunction in pre-eclampsia, and polymorphisms in Notch4 are associated with the severity of COVID-19–induced lung inflammation, thought to be mediated by endothelial activation (36, 37).

We found that Notch4 is expressed by the tumor endothelium in multiple types of tumors, which is supported by analysis of publicly available single-cell sequencing, which shows that Notch4 is significantly enriched in ECs across multiple subtypes of human breast cancer (16, 38). However, we observed tumors in which Notch4 was not expressed, LLC and MDA-MB-231, suggesting that endothelial response to tumors is heterogeneous. It is not clear what tumor-derived factors influence the endothelial expression of Notch4, although endothelial Notch4 is known to be upregulated by cortisol and downregulated by TNF, TGFβ, and IL10 (39, 40). In the seven tumor types we tested, we observed that there was complete concordance between endothelial expression of Notch4 and responsiveness to E7011/6-3-A6 despite expression of Notch4 in other cell types in some tumors. This suggests that endothelial expression is critical for anti-Notch4 treatment efficacy, which is an important consideration for therapeutic development. However, it is also possible that Notch4 expression on other cell types may contribute to or oppose the effects of endothelial Notch4. In particular, Notch4 expression on monocytes and fibroblasts, which play critical roles in tumorigenesis, has been shown to alter their function in a contact-dependent manner (bioRxiv 2023.10.24.563749). Further study is needed to dissect these possibilities, preferably using cell type–specific ablation of Notch4.

Another outstanding question relates to how Notch1 and Notch4 signaling overlaps in tumor ECs. Although we observed blockade of DLL4-induced activation of Notch4 proteolytic cleavage *in vitro*, our subsequent transcriptomic analysis did not observe inhibition of canonical Notch signaling by E7011/6-3-A6 treatment. Notch4 may signal through alternative pathways or may function in canonical Notch signaling transiently following initiation of anti-Notch4 treatment; both possibilities will be explored in future studies. The precise nature of the signals by which endothelial Notch4 regulates tumor endothelial function remains unclear. Additional translatomic analysis at various timepoints during tumor progression and single-cell RNA sequencing analysis may be needed to better understand endothelial and tumor microenvironmental influences of Notch4 on tumor growth.

In summary, we have generated a first-in-class antibody against Notch4 and documented the ability of Notch4 blockade to inhibit tumor growth. These findings support the potential for Notch4 blockade and specifically E7011 as candidate therapeutic approaches to treat cancer and alter the vasculature to improve tumor control. As our understanding of Notch4 signaling continues to expand, including its potential role in inflammation, combining E7011 with traditional chemotherapies and even immunotherapies, such as checkpoint inhibitors, may not only enhance their efficacy but also provide durable long-term treatment.

## Supplementary Material

Supplementary Table 1 List of constructs developed and used for experiments including Notch 1-4 expression constructs and the Notch luciferase reporter construct.

Supplementary Figure S1 A, Binding curves determined by SPR of 6-3-A6 and E7011 to murine and cynomolgus monkey Notch4. B, Binding of 6-3-A6 and E7011 at increasing concentrations to transfected HEK293 cell lines overexpressing full length human, murine, and cynomolgus Notch4 as assessed by mean fluorescent intensity (MFI) using flow cytometry.

Supplementary Figure S2 A-C, Weights of Balb/c nude mice implanted with Calu-6, SEKI and DU145 xenografts (n=5 for all tumor models) and treated with control IgG (all), 6-3-A6 (all), bevacizumab (SEKI), lenvatinib (SEKI), or paclitaxel (DU145). D, Mass of non-tumor bearing C57BL/6 mice treated IV with E7011 or control IgG twice weekly for 4 weeks (n=5). E, Representative images of the livers. Insets show 2x magnified view of the vessels at the periphery of the liver lobe. Scale bar = 6cm. F, Final tumor mass of orthotopically implanted Py8119 tumors treated IV with E7011 (25mg/kg) starting 12 hours after initial implantation (n=13 control IgG, n=12 E7011). Growth curve statistics performed with 2-way Anova, plotted as average ± SEM. Statistics by t-test, plotted as average ± SEM; *p<0.05.

Supplementary Figure S3 A, Schematic of experimental design of Py8119 orthotopically implanted tumors in RiboTag^EC^ mice. Female RiboTag^EC^ mice with 75mg/kg of tamoxifen (20mg/mL) for 5 days by IP injection. Py8119 tumors were implanted at 8 weeks of age and then treated once tumors reached 50mm3 with twice weekly IV control IgG or 6-3-A6 for a total of 10 days (n=3). B, Growth curve of RiboTagEC treated with 25mg/kg of IV control IgG or 6-3-A6 twice weekly for 10 days (n=3). C, Heatmap of endothelial expressed genes segregated by total mRNA input and ribosomal IP mRNA from control IgG treated and 6-3-A6 treated Py8119 tumors. D, PCA plots of the ribosomal IP fraction from control IgG and 6-3-A6 treated tumors. Two samples from the control IgG and 6-3-A6 groups were identified as outliers and removed from analysis. E, Heatmap of downstream Notch regulated genes from endothelial IP of the control IgG or 6-3-A6 treated tumors and quantitative rt-PCR of the canonical Notch target gene *Hes1* and *Rnd1* amplified from 6-3-A6 treated and control RiboTag IP fractions (n=4). F, Heatmaps of genes associated with significantly enriched or downregulated signaling pathways involved in inflammation and extracellular matrix interactions. All heatmap data presented as Z-scores. Statistics by t-test for qPCR studies

Supplementary Figure S4 A-B, Growth curves of subcutaneous B16F10 (n=3) and orthotopic Py8119 tumors (n=3) implanted into C57BL/6 mice and treated twice weekly IV with 25 m/kg of E7011 until tumors reached roughly 200mm3. Treatment duration was 4 days for B16F10 and 10 days for Py8119. C, Dose response curve of Calu-6 xenografts implanted subcutaneously in nude mice and treated with either control IgG, 1mg/kg, 3mg/kg or 10mg/kg of E7011 IV for 8 days (n=8). D, Representative images of CD31 staining and Hoescht dye staining following treatment. E, Quantitation of Hoescht+ stained area over total area. All quantitation were normalized to the control IgG. Statistics by 2-way ANOVA for growth curves and t-test for perfusion studies; *p<0.05, **p<0.01, ***p<0.001.
