# Correction: Inhibition of Notch4 Using Novel Neutralizing Antibodies Reduces Tumor Growth in Murine Cancer Models by Targeting the Tumor Endothelium

**DOI:** 10.1158/2767-9764.CRC-25-0695

**Published:** 2025-11-11

**Authors:** Jason W.-L. Eng, Yu Kato, Yusuke Adachi, Bhairavi Swaminathan, L.A. Naiche, Rahul Vadakath, Yoshimasa Sakamoto, Youya Nakazawa, Sho Tachino, Ken Ito, Takanori Abe, Yukinori Minoshima, Kana Hoshino-Negishi, Hideaki Ogasawara, Tomomi Kawakatsu, Miyuki Nishimura, Masahiko Katayama, Masashi Shimizu, Kazuhiro Tahara, Toshitaka Sato, Katsuhisa Suzuki, Kishan Agarwala, Masao Iwata, Kenichi Nomoto, Yoichi Ozawa, Toshio Imai, Yasuhiro Funahashi, Junji Matsui, Jan Kitajewski

In the original version of this article (1), the GEO accession number (GSE241630) listed in the Data Availability section, page 1884, is incorrect. It should be GSE241629. The number has been corrected in the latest online HTML and PDF versions of the article. The authors regret this error.
