# Supplementary figures and images for: Inhibition of Notch4 Using Novel Neutralizing Antibodies Reduces Tumor Growth in Murine Cancer Models by Targeting the Tumor Endothelium
Source: Cancer Res Commun. 2024 Jul 10;4(7):1881–93. doi: 10.1158/2767-9764.CRC-24-0081 (PMC11289863; doi:10.1158/2767-9764.CRC-24-0081)

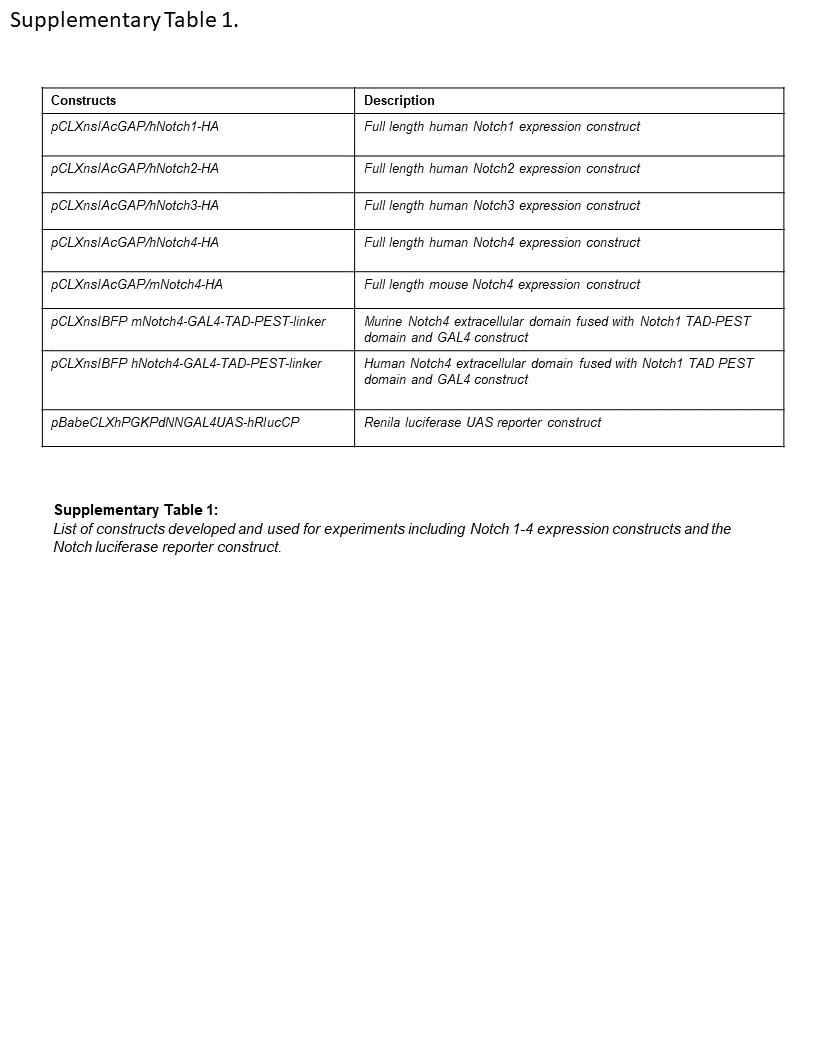

Supplement: Supplementary Table 1 — List of constructs developed and used for experiments including Notch 1-4 expression constructs and the Notch luciferase reporter construct. [file crc-24-0081_supplementary_table_1_suppst1.png]

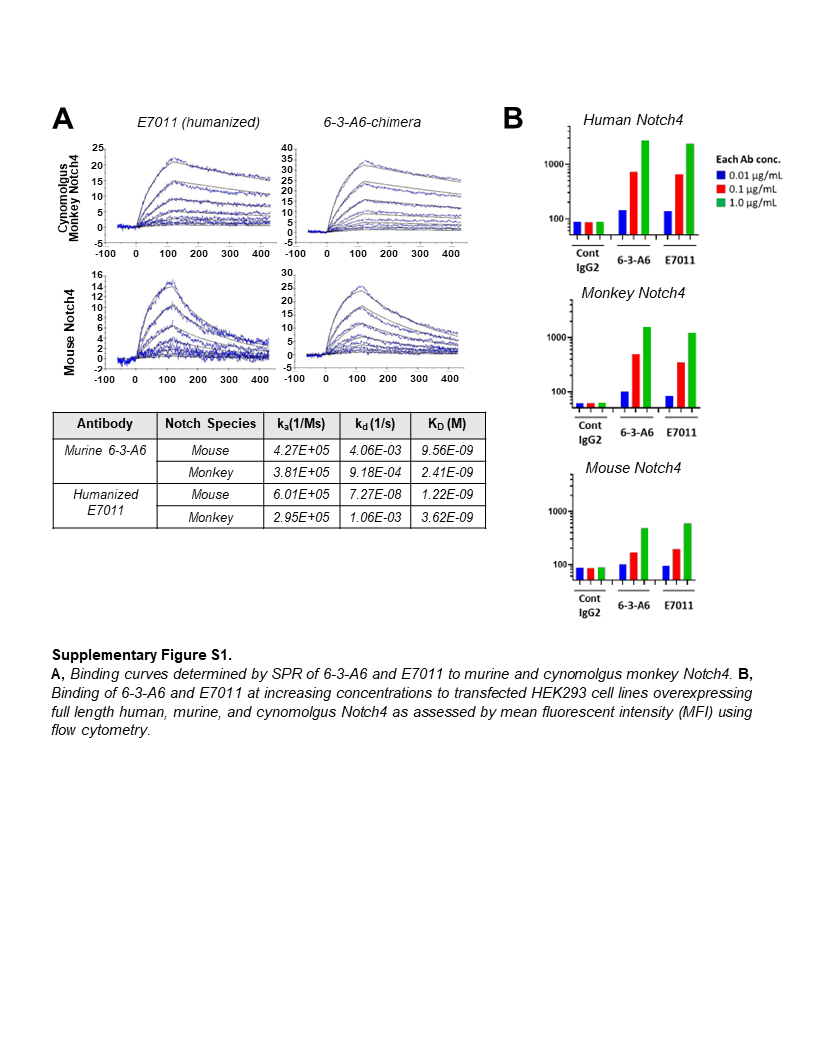

Supplement: Supplementary Figure S1 — A, Binding curves determined by SPR of 6-3-A6 and E7011 to murine and cynomolgus monkey Notch4. B, Binding of 6-3-A6 and E7011 at increasing concentrations to transfected HEK293 cell lines overexpressing full length human, murine, and cynomolgus Notch4 as assessed by mean fluorescent intensity (MFI) using flow cytometry. [file crc-24-0081_supplementary_figure_s1_suppsf1.png]

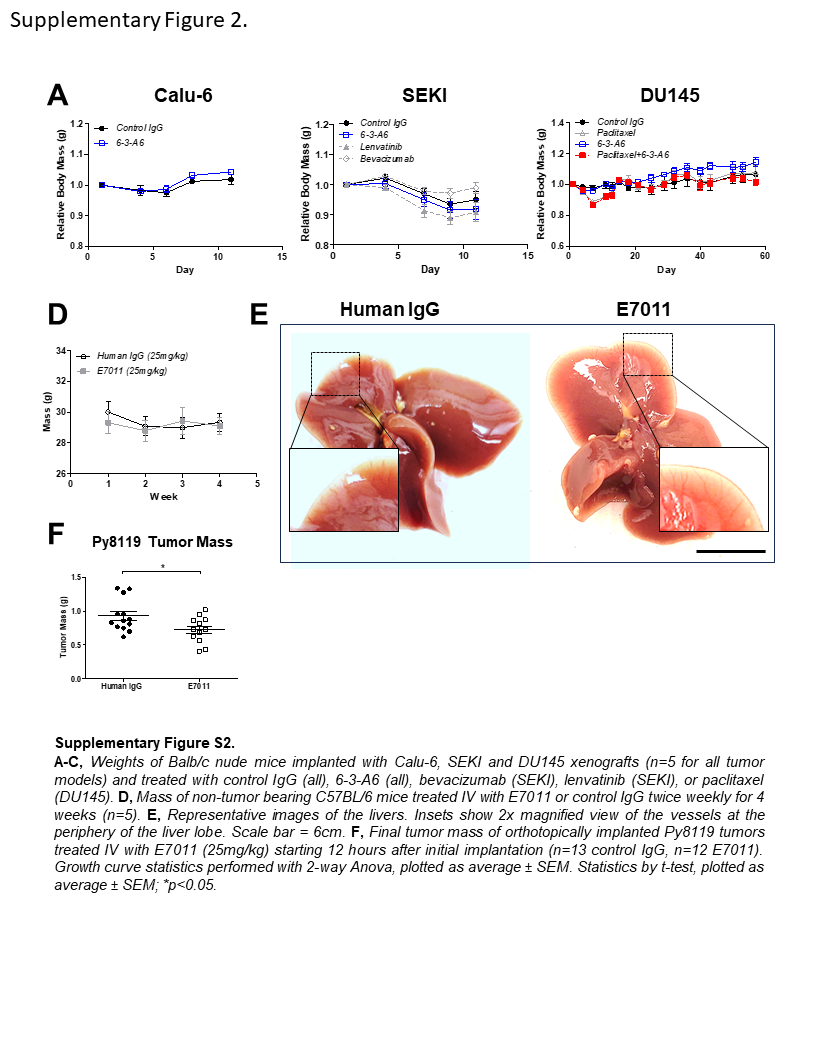

Supplement: Supplementary Figure S2 — A-C, Weights of Balb/c nude mice implanted with Calu-6, SEKI and DU145 xenografts (n=5 for all tumor models) and treated with control IgG (all), 6-3-A6 (all), bevacizumab (SEKI), lenvatinib (SEKI), or paclitaxel (DU145). D, Mass of non-tumor bearing C57BL/6 mice treated IV with E7011 or control IgG twice weekly for 4 weeks (n=5). E, Representative images of the livers. Insets show 2x magnified view of the vessels at the periphery of the liver lobe. Scale bar = 6cm. F, Final tumor mass of orthotopically implanted Py8119 tumors treated IV with E7011 (25mg/kg) starting 12 hours after initial implantation (n=13 control IgG, n=12 E7011). Growth curve statistics performed with 2-way Anova, plotted as average ± SEM. Statistics by t-test, plotted as average ± SEM; *p<0.05. [file crc-24-0081_supplementary_figure_s2_suppsf2.png]

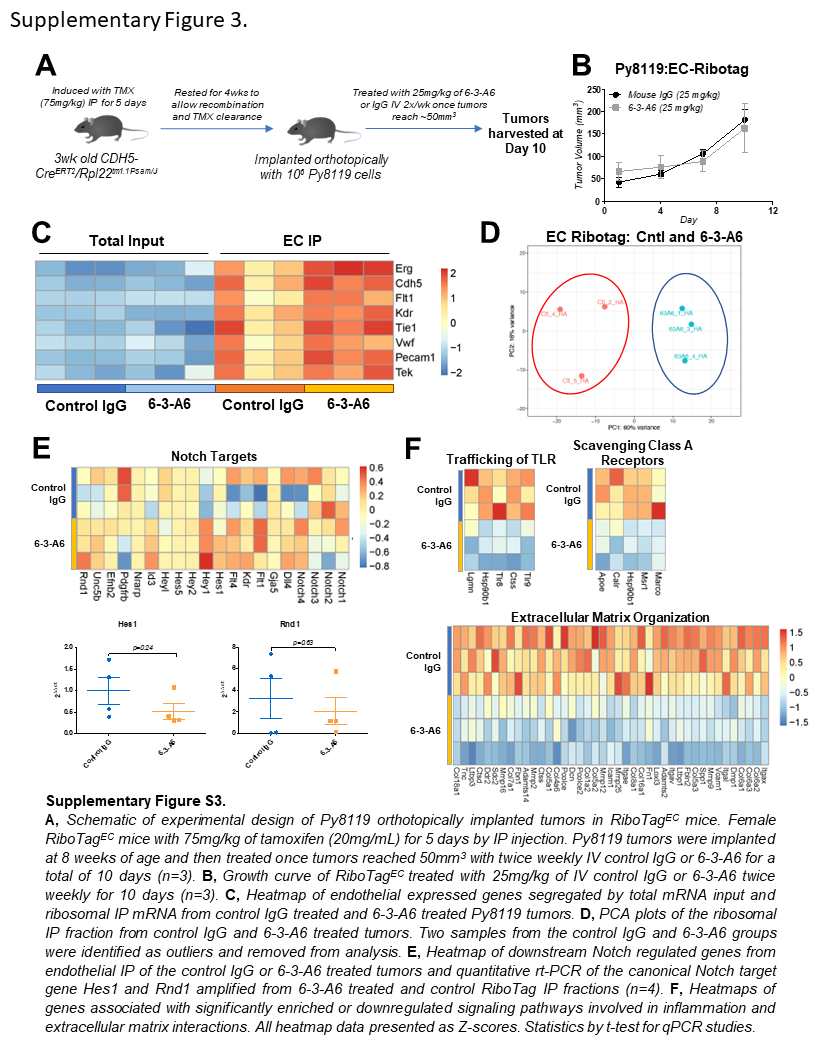

Supplement: Supplementary Figure S3 — A, Schematic of experimental design of Py8119 orthotopically implanted tumors in RiboTagEC mice. Female RiboTagEC mice with 75mg/kg of tamoxifen (20mg/mL) for 5 days by IP injection. Py8119 tumors were implanted at 8 weeks of age and then treated once tumors reached 50mm3 with twice weekly IV control IgG or 6-3-A6 for a total of 10 days (n=3). B, Growth curve of RiboTagEC treated with 25mg/kg of IV control IgG or 6-3-A6 twice weekly for 10 days (n=3). C, Heatmap of endothelial expressed genes segregated by total mRNA input and ribosomal IP mRNA from control IgG treated and 6-3-A6 treated Py8119 tumors. D, PCA plots of the ribosomal IP fraction from control IgG and 6-3-A6 treated tumors. Two samples from the control IgG and 6-3-A6 groups were identified as outliers and removed from analysis. E, Heatmap of downstream Notch regulated genes from endothelial IP of the control IgG or 6-3-A6 treated tumors and quantitative rt-PCR of the canonical Notch target gene Hes1 and Rnd1 amplified from 6-3-A6 treated and control RiboTag IP fractions (n=4). F, Heatmaps of genes associated with significantly enriched or downregulated signaling pathways involved in inflammation and extracellular matrix interactions. All heatmap data presented as Z-scores. Statistics by t-test for qPCR studies [file crc-24-0081_supplementary_figure_s3_suppsf3.png]

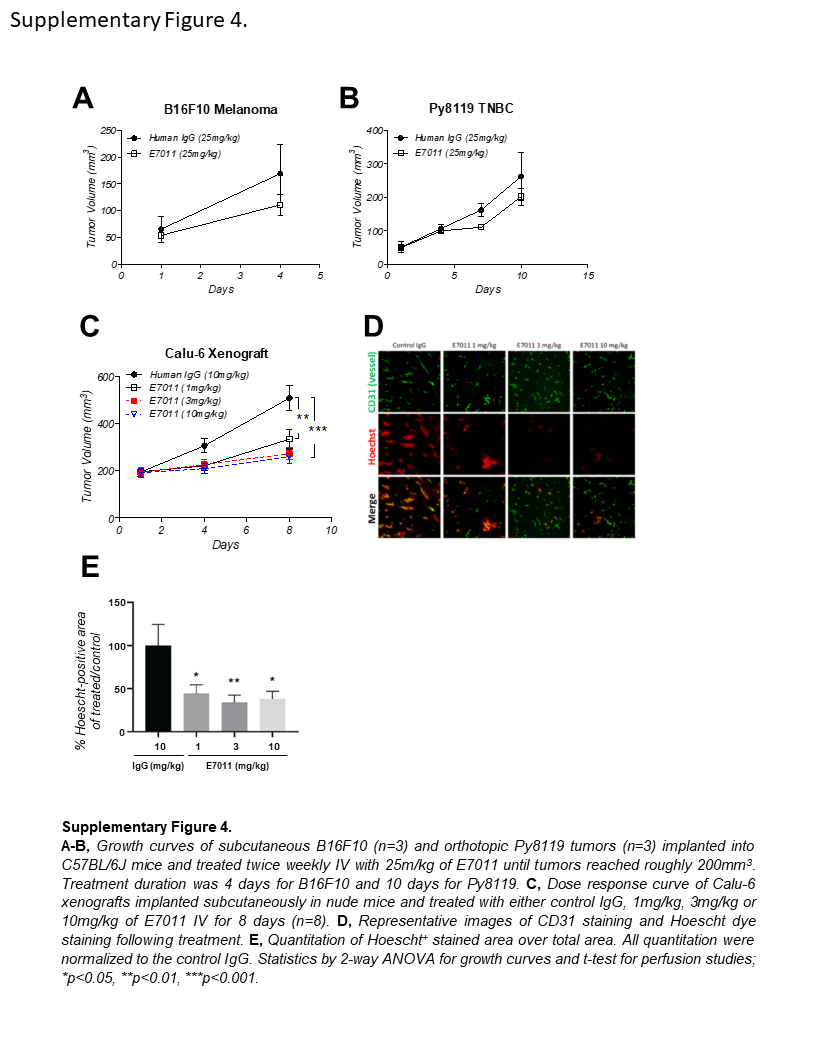

Supplement: Supplementary Figure S4 — A-B, Growth curves of subcutaneous B16F10 (n=3) and orthotopic Py8119 tumors (n=3) implanted into C57BL/6 mice and treated twice weekly IV with 25 m/kg of E7011 until tumors reached roughly 200mm3. Treatment duration was 4 days for B16F10 and 10 days for Py8119. C, Dose response curve of Calu-6 xenografts implanted subcutaneously in nude mice and treated with either control IgG, 1mg/kg, 3mg/kg or 10mg/kg of E7011 IV for 8 days (n=8). D, Representative images of CD31 staining and Hoescht dye staining following treatment. E, Quantitation of Hoescht+ stained area over total area. All quantitation were normalized to the control IgG. Statistics by 2-way ANOVA for growth curves and t-test for perfusion studies; *p<0.05, **p<0.01, ***p<0.001. [file crc-24-0081_supplementary_figure_s4_suppsf4.png]
